# Astrocyte-Secreted Factors Selectively Alter Neural Stem and Progenitor Cell Proliferation in the Fragile X Mouse

**DOI:** 10.3389/fncel.2016.00126

**Published:** 2016-05-18

**Authors:** Mary Sourial, Laurie C. Doering

**Affiliations:** ^1^McMaster Integrative Neuroscience Discovery and Study, McMaster University, HamiltonON, Canada; ^2^Pathology and Molecular Medicine, McMaster University, HamiltonON, Canada

**Keywords:** Fragile X syndrome, neural stem cells, neurospheres, astrocyte-secreted factors, *Fmr1* knockout mice, 2D DIGE

## Abstract

An increasing body of evidence indicates that astrocytes contribute to the governance and fine tuning of stem and progenitor cell production during brain development. The effect of astrocyte function in cell production in neurodevelopmental disorders is unknown. We used the Neural Colony Forming Cell assay to determine the effect of astrocyte conditioned media (ACM) on the generation of neurospheres originating from either progenitor cells or functional stem cells in the knock out (KO) Fragile X mouse model. ACM from both normal and *Fmr1*-KO mice generated higher percentages of smaller neurospheres indicative of restricted proliferation of the progenitor cell population in *Fmr1-*KO brains. Wild type (WT) neurospheres, but not KO neurospheres, showed enhanced responses to ACM from the *Fmr1*-KO mice. In particular, *Fmr1*-KO ACM increased the percentage of large neurospheres generated, representative of spheres produced from neural stem cells. We also used 2D DIGE to initiate identification of the astrocyte-secreted proteins with differential expression between *Fmr1-*KO and WT cortices and hippocampi. The results further support the critical role of astrocytes in governing neural cell production in brain development and point to significant alterations in neural cell proliferation due to astrocyte secreted factors from the Fragile X brain.

**Highlights:**

• We studied the proliferation of neural stem and progenitor cells in Fragile X.
• We examined the role of astrocyte-secreted factors in neural precursor cell biology.
• Astrocyte-secreted factors with differential expression in Fragile X identified.

## Introduction

Fragile X Syndrome (FXS) is the most common single-gene cause of autism spectrum disorder and intellectual impairment (Wang et al., 2012), with a frequency of about 1 in 7,100 males and 1 in 11,100 females (Hunter et al., 2014). FXS is associated with impairments in learning and memory, hyperactivity, hypersensitivity to sensory stimuli, increased susceptibility to seizures, and autistic behaviors (Penagarikano et al., 2007). The majority of FXS cases result from the epigenetic transcriptional silencing of the Fragile X Mental Retardation 1 gene (*FMR1*), which subsequently prevents the expression of fragile X mental retardation protein (FMRP) (Pieretti et al., 1991; Verkerk et al., 1991; Coffee et al., 1999, 2002; Eiges et al., 2007). FMRP is responsible for the translational regulation of many mRNAs (Miyashiro et al., 2003; Darnell et al., 2011; Ascano et al., 2012) including those involved in synaptic plasticity (Bassell and Warren, 2008; Darnell et al., 2011), and has been implicated in regulating the proliferation and differentiation of embryonic and adult neural precursor cells (NPCs) (Castrén et al., 2005; Eadie et al., 2009; Tervonen et al., 2009; Luo et al., 2010; Guo et al., 2011). A loss of FMRP and the associated memory and learning impairments observed in FXS, may therefore be linked to aberrant regulation of NPCs in the hippocampus.

There is a strong correlation between learning and memory capacity and hippocampal neurogenesis. A hallmark of the dentate gyrus (DG) of the hippocampal formation is lifelong neurogenesis to which NPCs contribute (Altman and Das, 1965). The enhanced plasticity of immature newborn neurons is suggested to enable learning and memory (Deng et al., 2010; Snyder and Cameron, 2012). In fact, many neurological diseases marked by cognitive decline such as Alzheimer’s, Parkinson’s, depression, and epilepsy, all show alterations in hippocampal neurogenesis (Zhao et al., 2008). Similarly, conditional knockout of FMRP in adult NPCs results in increased NPC proliferation, and the consequent impairment of hippocampus dependent learning (Guo et al., 2011). Notably, the majority of DG granule cells are born postnatally in the rodent brain (Altman and Bayer, 1990), and most adult born neurons are generated from early-born NPCs that reside in the DG as it is forming (Mathews et al., 2010). It is unknown whether early postnatal dysregulation of NPCs has ramifications on neurogenesis throughout life, or whether later interventions can correct abnormalities. Regulation of neurogenesis via FMRP is likely to impact early-born NPCs in the developing DG since expression of FMRP peaks at postnatal day 7 (Lu et al., 2004), which may underlie abnormal hippocampus-dependent memory in 3-week-old Fragile X mice (Bilousova et al., 2009).

The role of FMRP and its downstream effects have been largely limited to studies of neural populations in isolation. However, emerging evidence suggests that the glial environment and the role of FMRP in these cells are just as critical for proper brain development. Astrocytes for example, previously believed to be only support cells, secrete factors that actively regulate neurogenesis, neural function and communication (Barres, 2008). When cortical astrocytes devoid of FMRP are co-cultured with wild type (WT) neurons, the growth of the neurons is affected and normal synaptic formations among them are limited (Jacobs and Doering, 2010a). Of particular interest, the astrocytes within the hippocampus have been shown to promote NPC proliferation and neuronal differentiation (Song et al., 2002), and we hypothesized that a lack of FMRP in astrocytes may contribute to altered neural precursor/stem cell production.

To test this hypothesis, we used the Neural Colony Forming Cell (NCFC) Assay to examine the proliferation of neural stem cells within WT or Fragile X mice (*Fmr1-*KO) hippocampal neurospheres cultured in the presence of astrocyte conditioned media (ACM). The NCFC assay is specifically designed to distinguish between functional stem cells that form neurospheres more than 2 mm in diameter from neurospheres produced by the general precursor cell population with limited proliferative capacity (Louis et al., 2008). We found that Fragile X progenitor-derived neurospheres showed restricted proliferation in the presence of both WT and *Fmr1-*KO ACM. On the other hand, the proliferation of WT neural progenitor-derived neurospheres selectively decreased with *Fmr1-*KO ACM, while that of WT stem cell-derived neurospheres was enhanced. Interestingly, one population of WT neural progenitor-derived neurospheres showed decreased proliferation in the presence of cortical *Fmr1-*KO ACM, but not hippocampal *Fmr1-*KO ACM, highlighting a regional difference in astrocyte-secreted factors. Additionally, we compared the protein expression profiles of secreted factors from cortical and hippocampal astrocytes between WT and *Fmr1-*KO brains. Of the multiple proteins with differential expression that we detected, we identified the following proteins using mass spectrometry: multidrug resistance protein 1B, microphthalmia-associated transcription factor (MITF), haptoglobin, fasciculation, and elongation protein zeta 2, antithrombin III, and serum albumin. Together, these results indicate that signals derived from *Fmr1-*KO astrocytes affect the proliferation of WT neurospheres, and that *Fmr1-*KO neurospheres have intrinsic deficits in responding to environmental cues as evident in their indiscriminate response to WT versus *Fmr1-*KO ACM.

## Materials and Methods

### Animals

All animal experiments were performed in accordance with the guidelines set by the Canadian Council on Animal Care and were approved by the McMaster University Animal Research Ethics Board. The transgenic mouse colony was established from breeding pairs of FVB.129P2(B6)-*Fmr1^tm1Cgr^* mice obtained from Dr. Carl Dobkin at the New York State Institute for Basic Research in Developmental Disabilities (Staten Island, NY, USA). The WT and *Fmr1-*knockout (KO) mice were housed and maintained at the McMaster University Central Animal Facility.

### Neural Colony Forming Cell Assay

WT and *Fmr1-*KO postnatal day 1 (P1) mouse pups were decapitated and the brains processed for the NCFC Assay, using a modified protocol (Louis and Reynolds, 2010). Briefly, hippocampi were microdissected, minced into small pieces, and enzymatically digested for 20 min at 37°C (Pacey et al., 2006). The enzymes were inactivated by incubating the cell pellet for 10 min at 37°C in 4 mL of 1 mg/mL solution of trypsin inhibitor (Roche, Mississauga, ON, Canada) dissolved in NeuroCult complete NSC proliferation medium (StemCell Technologies, Vancouver, Canada). Cells were resuspended in 1 mL of NeuroCult complete NSC proliferation medium in the presence of 20 ng/mL epidermal growth factor (EGF, Sigma–Aldrich, St. Louis, USA), 10 ng/mL fibroblast growth factor-basic (FGF-2, Sigma–Aldrich), and 2 μg/mL heparin (Sigma–Aldrich). The cell suspension was passed through a 40-μm cell strainer and the cell density brought to a concentration of 650 cells/μL. For 2 replicates/culture, the following components were added in order: 1.7 mL NeuroCult NCFC Serum-Free Medium without cytokines, 330 μL NeuroCult NSC Proliferation Supplements (mouse), 6.6 μL EGF (of 10 μg/mL), 3.3 μL FGF-2 (of 10 μg/mL), 6.6 μL heparin (of 0.2%), 25 μL of cells at 650 cells/μL to a final density of 8,000 cells/35 mm culture dish, and 1.3 mL collagen solution. The neurosphere colonies were replenished with Complete NeuroCult Replenishment medium at 7 and 14 days *in vitro* (DIV) containing 0.5 μg/mL EGF, 0.25 μg/mL FGF, and 0.1 mg/mL heparin.

### Astrocyte-Conditioned Medium

Monolayers of cortical and hippocampal astrocytes were established (Jacobs and Doering, 2010b; Jones et al., 2012). P2 cortices of WT and *Fmr1-*KO mice were dissected and minced in Hank’s Buffered Salt Solution (Life Technologies, Burlington, ON, Canada) supplemented with HEPES (Life Technologies) and enzymatically digested in 0.25% trypsin (Life Technologies) supplemented with 1 mg/mL DNase (Roche). Enzymatic digestion of cells was inactivated by the addition of glial medium, which is comprised of minimal essential media (MEM) with Earl salts and L-glutamine (Life Technologies) supplemented with 10% horse serum (Life Technologies) and 0.6% glucose, and the cell suspension was passed through a 70 μm strainer. Cells were resuspended in glial medium and plated in T-75 cm^2^ flasks. Hippocampal astrocyte cultures were established using a modified protocol previously published (Jones et al., 2012). P2 hippocampi of WT and *Fmr1-*KO mice were dissected and minced in Hank’s Buffered Salt Solution supplemented with HEPES and mechanically digested in glial media of the same composition as that used for cortical cultures. Cells were resuspended in glial media and plated in T-75 cm^2^ flasks previously coated with 0.1 mg/mL Poly-D-Lysine Hydrochloride (Sigma–Aldrich) in 0.1 M borate buffer, pH 8.5. When hippocampal and cortical astrocytes reached 50% confluence, glial medium was switched to serum-free medium containing a final concentration of 1.25 ng/mL FGF-2 and no EGF (Pacey et al., 2006).

Astrocyte-conditioned media (ACM) was collected and concentrated 10X in concentrator tubes (Sartorius Vivaspin 20 Concentrator 5000 MWCO) at 3000 x*g* at 4°C. ACM was added to the NCFC assay when applicable at 1X concentration, and the concentrations of NeuroCult NCFC Serum-Free Medium, NeuroCult NSC Proliferation Supplements (mouse), EGF (10 μg/mL), FGF-2 (10 μg/mL), and heparin (0.2%) were adjusted to 9/10X at plating when ACM was used. Neurosphere colonies were cultured in different combinations listed in **Table 1**.

**Table 1:** Plating combinations of neurospheres and astrocyte conditioned media. Sample size (n) in parentheses.

Neurosphere genotype	Media condition
WT	No ACM (*n* = 4)
	WT cortical ACM (*n* = 4)
	WT hippocampal ACM (*n* = 3)
	*Fmr1-*KO cortical ACM (*n* = 5)
	*Fmr1-*KO hippocampal ACM (*n* = 5)
*Fmr1-*KO	No ACM (*n* = 5)
	WT cortical ACM (*n* = 5)
	WT hippocampal ACM (*n* = 5)
	*Fmr1-*KO cortical ACM (*n* = 5)
	*Fmr1-*KO hippocampal ACM (*n* = 5)


As a control, neurosphere proliferation was tested in the presence of 1X concentrated SFM containing 1.25 ng/mL FGF-2.

### Neurosphere Measurements

Neurosphere colonies were measured after 21 DIV on a 2 mm × 2 mm grid culture dish, using the 2X objective on the EVOS XL Core Cell Imaging System (Life Technologies). Images were obtained using the 4X objective. Individual neurospheres were classified into 4 categories based on diameter: ≤0.5 mm, 0.5–1 mm, 1–2 mm, ≥ 2 mm as previously documented (Louis and Reynolds, 2010). Analysis included WT (*n* = 9) and *Fmr1-*KO (*n* = 9) cultures without the addition of ACM, and with the addition of WT and *Fmr1-*KO cortical and hippocampal ACM in groups as shown in **Table 1**.

### Proteomic Analysis

Astrocyte conditioned media from WT and *Fmr1-*KO cortices and hippocampi were analyzed for protein expression using two-dimensional difference in gel electrophoresis (2D DIGE). ACM was 2D DIGE-analyzed by *Applied Biomics* (Hayward, CA, USA). Spots with the highest differential expression between WT and *Fmr1-*KO ACM as determined by 2D DIGE were selected for identification (4 spots from cortical ACM and 4 from hippocampal ACM, **Supplementary Tables S1** and **S2**). Protein identification was based on peptide fingerprint mass mapping (using MS data) and peptide fragmentation mapping (using MS/MS data).

### Statistical Analyses

**FIGURE 1:**
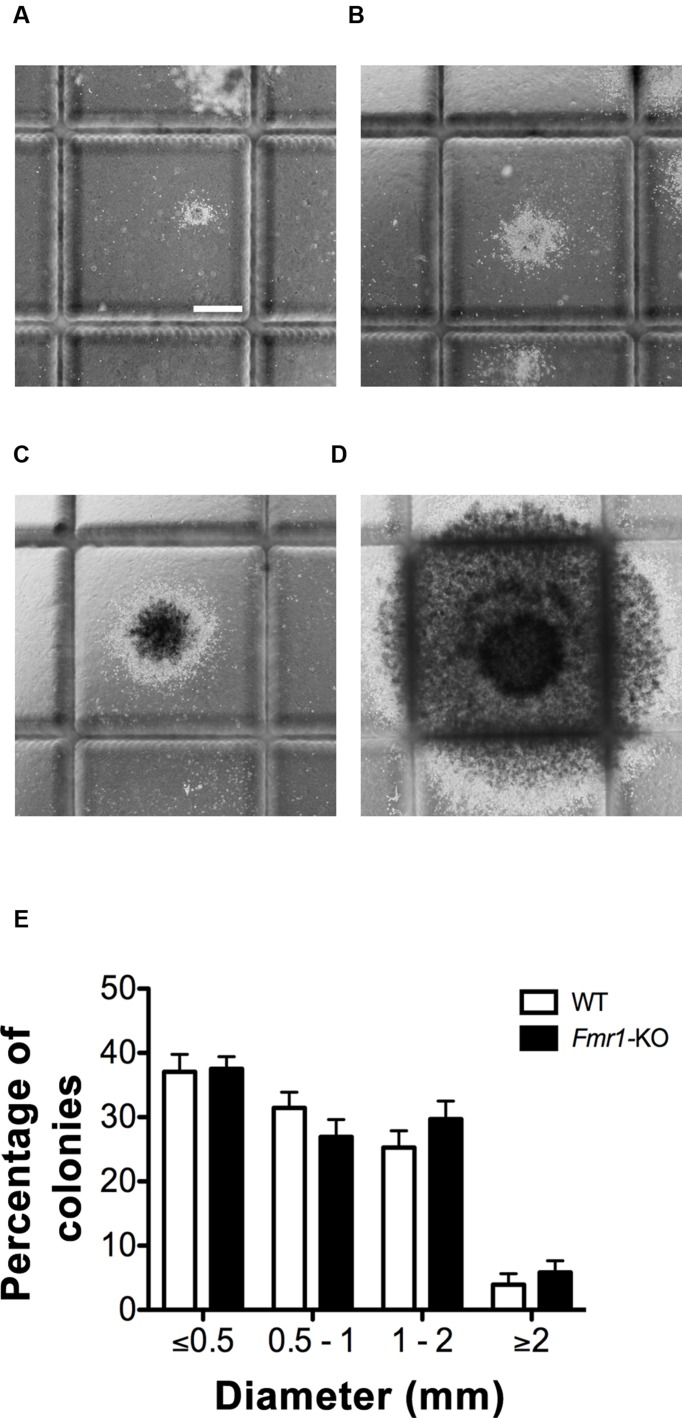
**Wild type (WT) and *Fmr1-*KO hippocampi generated four size categories of neurosphere colonies.** **(A)** Neurospheres ≤0.5 mm in diameter. **(B)** Neurospheres 0.5–1 mm in diameter. **(C)** Neurospheres 1–2 mm. **(D)** Neurospheres ≥2 mm. **(E)** No difference in the percentage of neurosphere colonies present between WT and *Fmr1-*KO cultures in each of the four size categories. Scale bar = 500 μm **(A–D)**. Abbreviations: WT, wild type; *Fmr1*-KO, *Fmr1* knockout.

**FIGURE 2:**
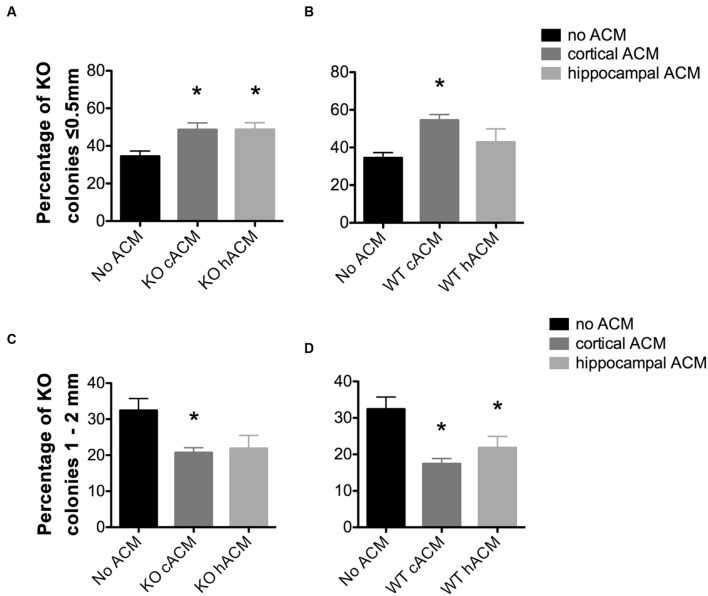
**Astrocyte conditioned media (ACM) decreased the size of *Fmr1-*KO progenitor-derived neurospheres.** **(A)** Both cortical and hippocampal ACM from *Fmr1*-KO astrocytes increased the percentage of KO neurospheres ≤0.5 mm relative to KO neurospheres without ACM (*p* = 0.014; *p* = 0.014, respectively). **(B)** WT cortical ACM also increased the percentage of KO neurospheres (*p* = 0.001) ≤0.5 mm relative to KO neurospheres without ACM. **(C)** Cortical *Fmr1-*KO ACM decreased the percentage of KO neurospheres 1–2 mm relative to KO neurospheres without ACM (*p* = 0.011), and hippocampal KO ACM resulted in a near significant decrease (*p* = 0.063). **(D)** Cortical and hippocampal WT ACM decreased the percentage of neurospheres 1–2 mm (*p* = 0.0031, *p* = 0.049) relative to neurospheres without ACM. ^∗^ denotes a significant difference compared to neurospheres with no ACM. Abbreviations: WT, wild type; *Fmr1*-KO, *Fmr1* knockout; ACM, astrocyte conditioned media.

**FIGURE 3:**
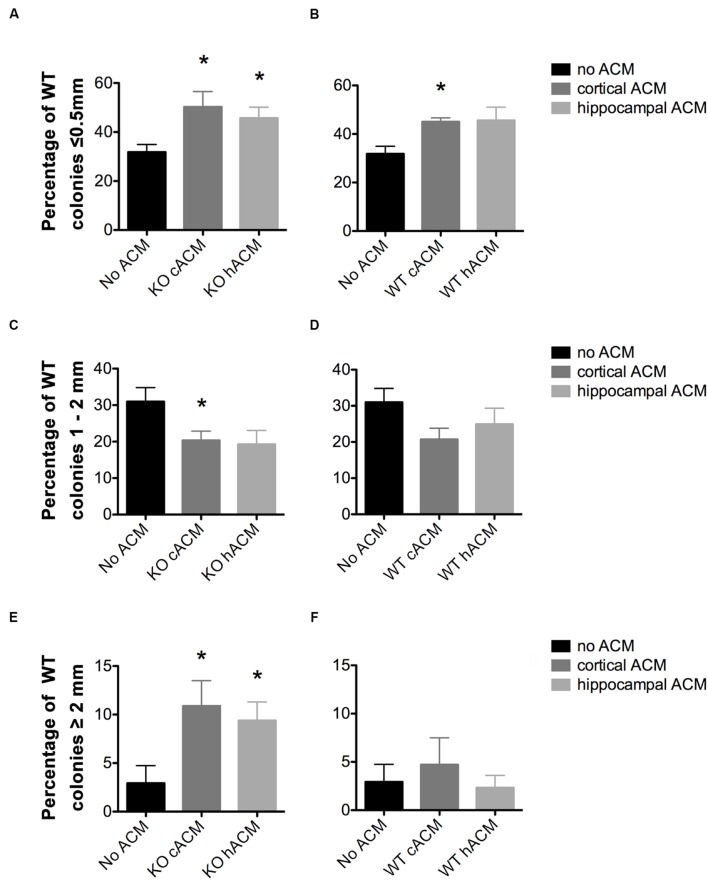
**WT neurospheres respond more selectively to *Fmr1-*KO ACM.** **(A)** Conditioned media from cortical and hippocampal KO astrocytes increased the percentage of neurospheres ≤0.5 mm relative to WT neurospheres without ACM (*p* = 0.046, *p* = 0.049, respectively). **(B)** WT Cortical ACM also increased the percentage of neurospheres ≤0.5 mm (*p* = 0.009) relative to WT neurospheres without ACM. WT hippocampal ACM caused a near significant increase in the percentage of WT neurospheres (*p* = 0.066). **(C)** *Fmr1-*KO cortical and hippocampal ACM decreased the percentage of WT neurospheres 1–2 mm (*p* = 0.049, *p* = 0.069, respectively). **(D)** No effect of WT ACM on WT neurospheres 1–2 mm in diameter. **(E)** Increased percentage of WT neurospheres ≥2 mm in diameter in the presence of cortical (*p* = 0.050) and hippocampal (*p* = 0.047) KO ACM relative to WT neurospheres without ACM. **(F)** No effect of WT ACM on WT neurospheres ≥2 mm in diameter. ^∗^ denotes significant difference from WT neurospheres with no ACM (*p <* 0.05). Abbreviations: WT, wild type; *Fmr1*-KO, *Fmr1* knockout; ACM, astrocyte conditioned media.

Statistical analysis was performed using GraphPad Prism (version 5.0). Two-way analysis of variance followed by Bonferroni’s *post hoc* test was used in **Figure 1**, and all other pairwise comparisons were done using a two-tailed Student’s *t*-test (**Figures 2** and **3**). Probabilities of *p* < 0.05 were considered significant. Data are expressed as group means and error bars represent the standard error of the mean (SEM).

## Results

### WT and *Fmr1*-KO Cultures Generate Similar Sizes of Neurospheres

The NeuroCult^®^ Neural Colony-Forming Cell (NCFC) Assay distinguishes stem from progenitor cell-derived neurospheres by generating clonally derived neurospheres in collagen that can be discriminated on the basis of size [(Louis et al., 2008); **Figures 1A–D**]. Neurosphere colonies larger than 2 mm in diameter contain cells with high proliferative potential and fulfill the functional criteria of stem cells (Louis et al., 2008). After 21 *DIV*, no differences were detected in the proportion of neurosphere colonies in each of the four size categories between WT and *Fmr1-*KO cultures (≤0.5 mm, 0.5–1 mm, 1–2 mm, ≥2 mm diameter, **Figure 1E**). However, the larger the neurosphere size category, the fewer the percentage of neurospheres in that category regardless of genotype (*F*_3,64_ = 68.16, *p* < 0.0001), as expected since fewer NPCs have an increased proliferative capacity (Reynolds and Rietze, 2005). Compared to neurospheres ≤0.5 mm in diameter, there were 86.9% fewer neurospheres with a diameter ≥2 mm. Together, these results indicated that while there were significantly more neurospheres with a lower proliferative potential, lack of FMRP did not affect the distribution of neurospheres in the 4 size categories after 21 *DIV* in our culture system, and thus the proportion of stem or progenitor cell-derived neurospheres was not different when comparing the two genotypes.

### ACM Modulates the Size of *Fmr1*-KO Neurospheres

Our previous work has shown that signals from Fragile X astrocytes contribute to the immature neuronal morphology characteristic of Fragile X (Jacobs and Doering, 2010a; Jacobs et al., 2010), and so we examined whether Fragile X astrocyte-secreted factors could affect the proliferative capacity of neurospheres. We collected ACM from primary astrocyte cultures and added it to cultured NPCs in collagen as outlined (**Table 1**). When testing for variances between groups, we found a significant interaction between ACM conditions and *Fmr1-*KO neurosphere size (*F*_12,80_ = 2.69, *p* = 0.0042). ACM did not affect the total number of *Fmr1-*KO colonies generated (**Supplementary Figure S1**).

A larger proportion of *Fmr1*-KO neurospheres measured ≤0.5 mm in parallel to a smaller proportion of neurospheres that were 1–2 mm, in the presence of WT and *Fmr1*-KO ACM. Specifically, there was an increase in the percentage of small neurospheres (≤0.5 mm) in *Fmr1*-KO cultures (34.51 ± 2.76%) with the application of ACM from the *Fmr1*-KO cortex (48.62 ± 3.59%, *p* = 0.014; **Figure 2A**), *Fmr1*-KO hippocampus (48.77 ± 3.59%, *p* = 0.014, **Figure 2A**), and WT cortex (54.48 ± 3.01%, *p* = 0.001, **Figure 2B**). At the same time, application of ACM from the *Fmr1*-KO cortex (20.73 ± 1.35%, *p* = 0.011, **Figure 2C**), WT cortex (17.43 ± 1.42%, *p* = 0.0031, **Figure 2D**), and WT hippocampus (21.83 ± 3.13%, *p* = 0.049, **Figure 2D**) caused a decrease in the percentage of larger neurospheres (1–2 mm) relative to *Fmr1*-KO neurospheres without ACM (32.41 ± 3.30%). Interestingly, ACM did not affect the percentages of *Fmr1-*KO neurospheres measuring 0.5–1 mm or ≥2 mm (**Supplementary Figure S2**). These results demonstrated that astrocyte secreted factors decreased *Fmr1-*KO neurosphere size, suggesting that WT and *Fmr1*-KO ACM, from the cortex and the hippocampus, restricted the proliferation of progenitor-derived neurospheres.

### WT Neurospheres Have an Enhanced Sensitivity to *Fmr1*-KO ACM

The addition of ACM significantly affected the generation of WT neurospheres of different sizes (*F*_12,64_ = 2.69, *p* = 0.0013). This is evident from the reduced total number of WT colonies generated in the presence of ACM from cortical *Fmr1-*KO astrocytes (**Supplementary Figure S1**). In addition, the percentage of WT neurospheres measuring ≤0.5 mm in diameter increased in the presence of both WT and *Fmr1-*KO ACM. ACM from the *Fmr1-*KO cortex (50.24 ± 6.28%, *p* = 0.046, **Figure 3A**), *Fmr1-*KO hippocampus (45.66 ± 1.59%, *p* = 0.049, **Figure 3A**), and WT cortex (45.04 ± 1.59%, *p* = 0.009; **Figure 3B**) increased the percentage of WT neurospheres ≤0.5 mm relative to WT neurospheres without ACM (31.83 ± 3.09%). Notably, ACM from WT astrocytes had no effect on WT neurospheres measuring 0.5–1 mm, 1–2 mm (**Figure 3D**), or ≥2 mm (**Figure 3F**).

On the other hand, ACM from the *Fmr1*-KO cortex (20.31 ± 2.58%, *p* = 0.049, **Figure 3C**) and *Fmr1*-KO hippocampus (19.26 ± 3.78%, *p* = 0.069, **Figure 3C**) reduced the percentage of WT neurospheres 1–2 mm diameter compared to WT neurospheres without ACM (30.97 ± 3.86%). Interestingly, only ACM from the *Fmr1*-KO cortex (18.58 ± 3.56%, *p* = 0.012, **Supplementary Figure S2**) decreased the percentage of WT neurospheres measuring 0.5–1 mm compared to those without ACM (34.27 ± 2.71%), potentially due to the genotype-region mismatch of *Fmr1*-KO cortical ACM and WT hippocampal neurospheres (data not shown).

### *Fmr1*-KO ACM Increases Stem Cell Production in WT Neurospheres

In sharp contrast, the percentage of WT neurospheres ≥2 mm, originating from functional stem cells, increased in the presence of ACM from the *Fmr1*-KO cortex (10.88 ± 2.62%, *p* = 0.050; **Figure 3E**) and the *Fmr1*-KO hippocampus (9.40 ± 1.92%, *p* = 0.047; **Figure 3E**) in comparison to cultures without ACM (2.93 ± 1.81%). The proportion of neurospheres ≥2 mm did not change in the presence of ACM from WT astrocytes from the cortex (4.71 ± 2.78%, *p* = 0.61; **Figure 3F**) or the hippocampus (2.31 ± 1.29%, *p* = 0.81 **Figure 3F**). Taken together, the results showed that *Fmr1*-KO ACM but not WT ACM significantly affected WT neurosphere size. This suggests that *Fmr1*-KO astrocytes provide aberrant signals in the control of NPC proliferation.

### Differential Expression of Astrocyte-Secreted Proteins in Fragile X

**FIGURE 4:**
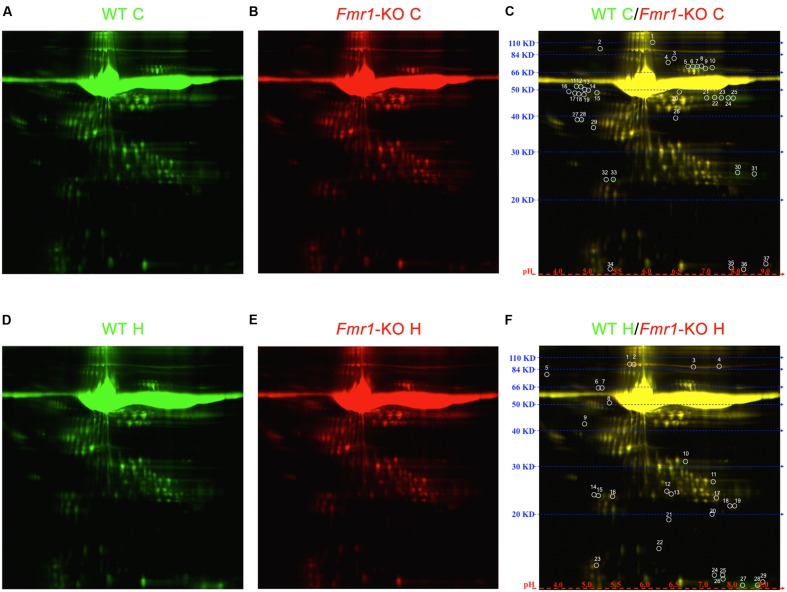
**2D DIGE of cortical and hippocampal ACM.** Proteome map of ACM from **(A)** WT cortical samples or **(B)** *Fmr1-*KO cortical samples. **(C)** 37 spots showed at least 50% expression difference in ACM from *Fmr1-*KO cortices relative to their WT counterparts. Proteome map of ACM from **(D)** WT hippocampal samples or **(E)** *Fmr1-*KO hippocampal samples. **(F)** 29 spots showed at least 50% expression difference in ACM from *Fmr1-*KO hippocampi. Abbreviations: WT, wild type; *Fmr1*-KO, *Fmr1* knockout; ACM, astrocyte conditioned media; C, cortical; H, hippocampal; 2D DIGE, two-dimensional difference in gel electrophoresis.

Proteomic analyses were performed on ACM to determine the identity of secreted proteins and compare between WT and *Fmr1-*KO astrocytes Data Sheet. 2D DIGE of spots that were at least expressed at ± 1.5 fold in *Fmr1-*KO compared to WT samples revealed 37 spots in cortical ACM (**Figures 4A–C**) and 29 spots in hippocampal ACM (**Figures 4D–F**). Eight of the spots were identified by mass spectrometry as: multidrug resistance protein 1B, MITF, haptoglobin, fasciculation, and elongation protein zeta 2 (FEZ2), antithrombin III, and serum albumin (**Table 3**). Three spots corresponded to serum albumin, which were not immediately adjacent on the gel possibly due to post-translational modifications or disintegration. Notably, the expression of the majority of astrocyte-secreted proteins from the *Fmr1*-KO cortex was downregulated relative to WT (34 out of 37 spots, **Supplementary Table S1**), whereas most secreted proteins from the *Fmr1*-KO hippocampus were upregulated compared to WT (18 out of 29 spots, **Supplementary Table S2**). Differences between cortical and hippocampal astrocyte-secreted factors again confirm the heterogeneity of astrocytes.

## Discussion

In this study, we examined potential differences in the proportions of neural progenitor and neural stem cells between WT and *Fmr1*-KO NPCs *in vitro* using the NCFC Assay. Our results confirmed that the NCFC Assay generates four size categories of neurospheres, in turn reflecting different proliferative potentials in the NPC population (Louis et al., 2008). We also found that *Fmr1-*KO neural progenitor cells had a non-autonomous cell deficit in correctly responding to astrocyte secreted factors. This was evident in the indiscriminate response of *Fmr1-*KO neurospheres to ACM from WT or *Fmr1-*KO astrocytes, which contrasted with the specific response of WT neurospheres to *Fmr1-*KO, but not WT, ACM (**Table 2**). Interestingly, *Fmr1-*KO ACM increased the proliferation of WT functional neural stem cells, highlighting the abnormal signals that astrocytes send to NPCs in the absence of FMRP.

**Table 2:** Summary of results.

	Neurosphere Size	WT ACM	KO ACM
WT Neurospheres	≤0.5 mm	Δ↑	Δ↑
	0.5–1 mm	no Δ	Δ↓
	1–2 mm	no Δ	Δ↓
	≥2 mm	noΔ	Δ↑
KO Neurospheres	≤0.5 mm	Δ↑	Δ↑
	0.5–1 mm	no Δ	no Δ
	1–2 mm	Δ↓	Δ↓
	≥2 mm	noΔ	no Δ


**Table 3:** Identity of proteins in ACM.

Spot number	Gel	Protein	Fold difference (*Fmr1-*KO/WT)	Theoretical PI/MW	Protein score C.I. (%)	Total ion C.I. (%)
2	1 (cortical ACM)	Multidrug resistance protein 1B	-4.9	8.5/140,905	0	97
16		Microphthalmia-associated transcription factor (MITF)	-4.8	5.9/58,590	90	100
28		Haptoglobin	-10.6	5.9/38,728	100	100
33		Fasciculation and elongation protein zeta-2 (FEZ2)	-12.9	4.5/39,076	99	91
8	2 (hippocampal ACM)	Antithrombin-III	2.4	6.1/51,971	100	100
25		Serum albumin	-7.0	5.8/68,648	100	100
27		Serum albumin	-25.7	5.8/68,648	100	100
28		Serum albumin	-45.3	5.8/68,648	100	100


NPCs comprise populations of neural stem and progenitor cells. Neural stem cells self-renew and are multipotent, giving rise to all neural lineages, namely neurons, astrocytes, and oligodendrocytes. Neural progenitor cells, on the other hand, have limited proliferative capacity and restricted potential in differentiating to distinct lineages. Previous research has reported enhanced NPC proliferation in the embryonic SVZ (Castrén et al., 2005), the embryonic VZ and SVZ combined (Tervonen et al., 2009), and the adult DG (Luo et al., 2010; Guo et al., 2011) in association with lack of FMRP. Given this, we expected to observe differences between WT and *Fmr1*-KO cultures in the proportions of neural progenitor- or stem cell-derived neurospheres generated. Our results alternatively show that there were in fact no inherent differences between the proliferative propensities of the neurospheres of each genotype, in line with Eadie et al. (2009), who reported no differences in the proliferation of NPCs in the DG of adult *Fmr1*-KO mice *in vivo* (Eadie et al., 2009). It is noteworthy to mention that our study is the first to examine hippocampal NPCs from the *Fmr1-*KO mouse brain on an FVB genetic background. While we detected no differences in neural progenitor and stem cell proliferation in our culture system between WT and *Fmr1*-KO cultures after 21 *DIV*, differences may exist at other time points especially since FMRP is developmentally regulated (Till et al., 2012). Till et al. (2012) indicate that *Fmr1*-KO mice show delays in cortical development at time points that coincide with the highest FMRP levels in normal brains. Notably, astrocytes of the developing hippocampus express FMRP (Pacey and Doering, 2007), suggesting that astrocytes contribute to the hippocampal impairments seen in Fragile X.

We identified a subset of astrocyte-secreted factors whose expression was up- or down-regulated by at least 50% in *Fmr1*-KO samples. These included multidrug resistance protein 1B, MITF, haptoglobin, and FEZ2 in cortical ACM, and antithrombin III and serum albumin in hippocampal ACM. These factors were all downregulated with the exception of antithrombin III (**Supplementary Tables S1** and **S2**). Notably, the expression of multidrug resistance protein 1 has been reported in human NPCs where it promotes the proliferation of NPCs (Yamamoto et al., 2009). Thus, our finding of decreased multidrug resistance protein 1 expression in *Fmr1-*KO cortical ACM is in agreement with the decreased neural progenitor proliferation detected in the presence of *Fmr1-*KO cortical ACM. Unlike the multidrug resistance family of proteins, the function of the FEZ family of proteins is elusive. Previous research has found that FEZ2 interacts with 59 proteins in categories that include transcription, translation, apoptosis, signal transduction, neuronal cell development, and cytoskeleton and centrosome (Alborghetti et al., 2011). The function of FEZ2 remains poorly understood at this time. The expression of albumin and antithrombin III in the developing brain under normal conditions suggests that they play an important role in brain development (Dziegielewska et al., 1984; Deschepper et al., 1991). In fact, astrocytes of the developing brain have been shown to secrete antithrombin III in culture, similar to our findings (Deschepper et al., 1991). Albumin is also necessary for astrocytes to provide energy to the developing brain (Tabernero et al., 1999) and to synthesize oleic acid (Tabernero et al., 2001). In turn, oleic acid promotes neuronal differentiation (Tabernero et al., 2001). Given that astrocytes show developmental delays in FXS (Jacobs et al., 2010), we suggest that the expression levels of albumin and antithrombin III that we detected in *Fmr1*-KO hippocampal ACM may mirror the expression levels in ACM from the WT hippocampus at earlier time points. We are currently studying the role of the astrocyte secreted factors in Fragile X in order to better understand the extent of astrocyte involvement in Fragile X and associated neurodevelopmental disorders.

Astrocyte-secreted factors, such as thrombospondin-1 (Lu and Kipnis, 2010), fibroblast growth factor-2 (Kirby et al., 2013), and clusterin (Cordero-Llana et al., 2011) promote NPC proliferation, neurogenesis, or both. Indeed, astrocytes are an important component of the hippocampal neurogenic niche (Song et al., 2002). Our previous work has shown that signals from *Fmr1*-KO astrocytes can alter the development and growth of co-cultured WT neurons, an effect not seen with WT astrocytes (Jacobs and Doering, 2010a). Thus, we hypothesized that ACM from WT astrocytes would correct aberrant proportions of progenitor and stem cell-derived *Fmr1*-KO neurospheres, whereas *Fmr1*-KO ACM would alter the proportions of each of the WT neurosphere size categories. Our results demonstrated that ACM from *Fmr1*-KO astrocytes did alter the proliferation of WT neural progenitor and functional stem cells, restricting the proliferation of neural progenitors while increasing the proliferation of neural stem-derived neurospheres. It remains to be determined why *Fmr1-*KO ACM exerts opposite effects on neural progenitor compared to neural stem cells.

Intriguingly, the percentage of WT neurospheres measuring ≤0.5 mm increased in the presence of WT and *Fmr1*-KO ACM from the cortex and the hippocampus, similar to *Fmr1*-KO neurospheres of the same size category. These neurospheres have a limited ability for self-renewal (Louis et al., 2008). The generic response of WT neurospheres to ACM is interesting, as it was only detected in this size category, and mirrors the effect of ACM on *Fmr1*-KO neurospheres. This suggests a non-cell autonomous response of WT NPCs of limited potential as demonstrated by their generic response to astrocyte-secreted factors regardless of FMRP expression.

Lack of FMRP caused a defect in *Fmr1*-KO NPCs to respond correctly to environmental cues. *Fmr1*-KO neurospheres showed an indiscriminate response toward WT and *Fmr1*-KO ACM. We found that ACM from WT and *Fmr1*-KO astrocytes caused a global increase in the proportion of *Fmr1*-KO neurospheres ≤0.5 mm. In parallel, ACM decreased the proportion of *Fmr1*-KO neurospheres 1–2 mm in diameter relative to *Fmr1*-KO neurospheres of the same size without ACM. Collectively, this indicates that ACM restricted the proliferation of *Fmr1-*KO neural progenitors without affecting the proliferation of stem cells. The fact that the percentages of some *Fmr1*-KO neurosphere size categories (i.e., 0.5–1 mm and ≥2 mm) did not change in the presence of ACM suggests that not all NPC populations are targets of astrocyte-secreted factors in Fragile X.

In contrast to *Fmr1*-KO NPCs, only ACM from *Fmr1*-KO astrocytes decreased the percentage of WT neurospheres 1–2 mm, and increased that of neurospheres ≥2 mm in diameter. The selective response of WT neurospheres to *Fmr1*-KO ACM points to a non-cell autonomous defect in NPC proliferation in the absence of FMRP caused by abnormal *Fmr1-*KO astrocytic cues. This was particularly evident in the effect of ACM on neurospheres ≥2 mm in diameter, where the percentage of WT neurospheres was increased in the presence of *Fmr1*-KO ACM, while that of *Fmr1*-KO neurospheres was not. This population of neurospheres represents multipotent and proliferative functional stem cells (Louis et al., 2008). The apparent aberrant signaling of *Fmr1*-KO astrocytes may be due to compensatory measures for the lack of responsiveness of *Fmr1*-KO neurospheres to cellular or environmental cues.

The addition of *Fmr1*-KO cortical ACM to WT neurospheres caused the most robust changes, affecting the total number of neurospheres generated (**Supplementary Figure S1**) as well as the percentages of neurospheres in all size categories relative to WT neurospheres without ACM (**Supplementary Figure S2**), which highlights the heterogeneity of astrocytes (Matyash and Kettenmann, 2010). This is likely due to the genotypic-spatial mismatch between hippocampal WT neurospheres and cortical *Fmr1*-KO ACM. Indeed, of all combinations tested, ACM from *Fmr1*-KO cortical astrocytes decreased the percentage of WT colonies measuring 0.5–1 mm. Thus, the proliferation of NPCs that make up colonies 0.5–1 mm is not solely changed by astrocyte-secreted factors from Fragile X brains. Rather, the enhanced effect of *Fmr1*-KO cortical ACM on WT hippocampal neurospheres resulted in our finding, which seems to only apply to neurospheres 0.5–1 mm. In agreement with the specificity of astrocytes, hippocampal astrocytes are shown to promote NPC proliferation and neurogenesis, whereas spinal cord astrocytes do not (Song et al., 2002). Similarly, ACM from the hippocampus increases the number of neurons differentiated from human NPCs, whereas cortical ACM does not (Cordero-Llana et al., 2011).

One of the functions of hippocampal neurogenesis is learning and memory (Deng et al., 2010). We thus hypothesized that impaired learning, characteristic of FXS, is attributed to aberrant NPC proliferation and neurogenesis. *Fmr1-*KO mice have demonstrated deficits in hippocampus-dependent learning such as exaggerated inhibitory avoidance extinction (Dölen et al., 2007) and trace conditioning tasks (Hayashi et al., 2007). Interestingly, conditional ablation of FMRP from adult NPCs results in learning deficits on the trace conditioning and delayed non-matching-to-place radial arm maze tasks, both of which require an intact hippocampus, and conditional restoration of FMRP results in improved performance on these tasks (Guo et al., 2011). Therefore, functional restoration of hippocampal neurogenesis may alleviate learning and memory impairments in Fragile X.

## Summary

In this study, we examined the proliferation of hippocampal neural progenitor and stem cells from the early postnatal *Fmr1-*KO mouse brain. We found that *Fmr1-*KO progenitor-derived neurospheres showed decreased proliferation in response to WT and *Fmr1-*KO ACM, thereby demonstrating non-cell autonomous defects in responding correctly to astrocyte-secreted factors. In contrast, WT neurospheres showed a specific response to *Fmr1-*KO ACM, where *Fmr1-*KO ACM restricted the proliferation of neural progenitors, and enhanced the proliferation of stem cells. We also identified some of the astrocyte secreted factors with ± 1.5 fold expression difference in *Fmr1-*KO brains. To our knowledge, this is the first time that astrocyte-secreted factors are assayed and a number of them identified with mass spectrometry in relation to Fragile X. In turn, this work opens up new possibilities in investigating the functions of these factors and uncovering the role of astrocytes in Fragile X and neurodevelopmental disorders.

## Author Contributions

MS conception and design, collection and/or assembly of data, data analysis and interpretation, manuscript writing, final approval of manuscript. LD conception and design, financial support, provision of study material or patients, data analysis and interpretation, manuscript writing, final approval of manuscript.

## Conflict of Interest Statement

The authors declare that the research was conducted in the absence of any commercial or financial relationships that could be construed as a potential conflict of interest.

## Supplementary Material

**FIGURE S1 | Total number of colonies generated by the addition of ACM to WT and *Fmr1-*KO neurospheres.** Abbreviations: WT, wild type; KO, *Fmr1* knockout; ACM, astrocyte conditioned media; c, cortical; h, hippocampal.

**FIGURE S2 | Summary of the effect of astrocyte conditioned media (ACM) on neurosphere size.** **(A)** WT neurospheres, and **(B)** *Fmr1-*KO neurospheres. ^∗^ denotes significant difference from neurospheres with no ACM (*p* < 0.05). Abbreviations: WT, wild type; *Fmr1-*KO, *Fmr1* knockout; ACM, astrocyte conditioned media.

**TABLE S1 | Spots from cortical ACM with at least 1.5 fold difference relative to WT.**

**TABLE S2 | Spots from hippocampal ACM with at least 1.5 fold difference relative to WT.**
