# Supplementary figures and images for: Astrocyte-Secreted Factors Selectively Alter Neural Stem and Progenitor Cell Proliferation in the Fragile X Mouse
Source: Front Cell Neurosci. 2016 May 18;10:126. doi: 10.3389/fncel.2016.00126 (PMC4870401; doi:10.3389/fncel.2016.00126)

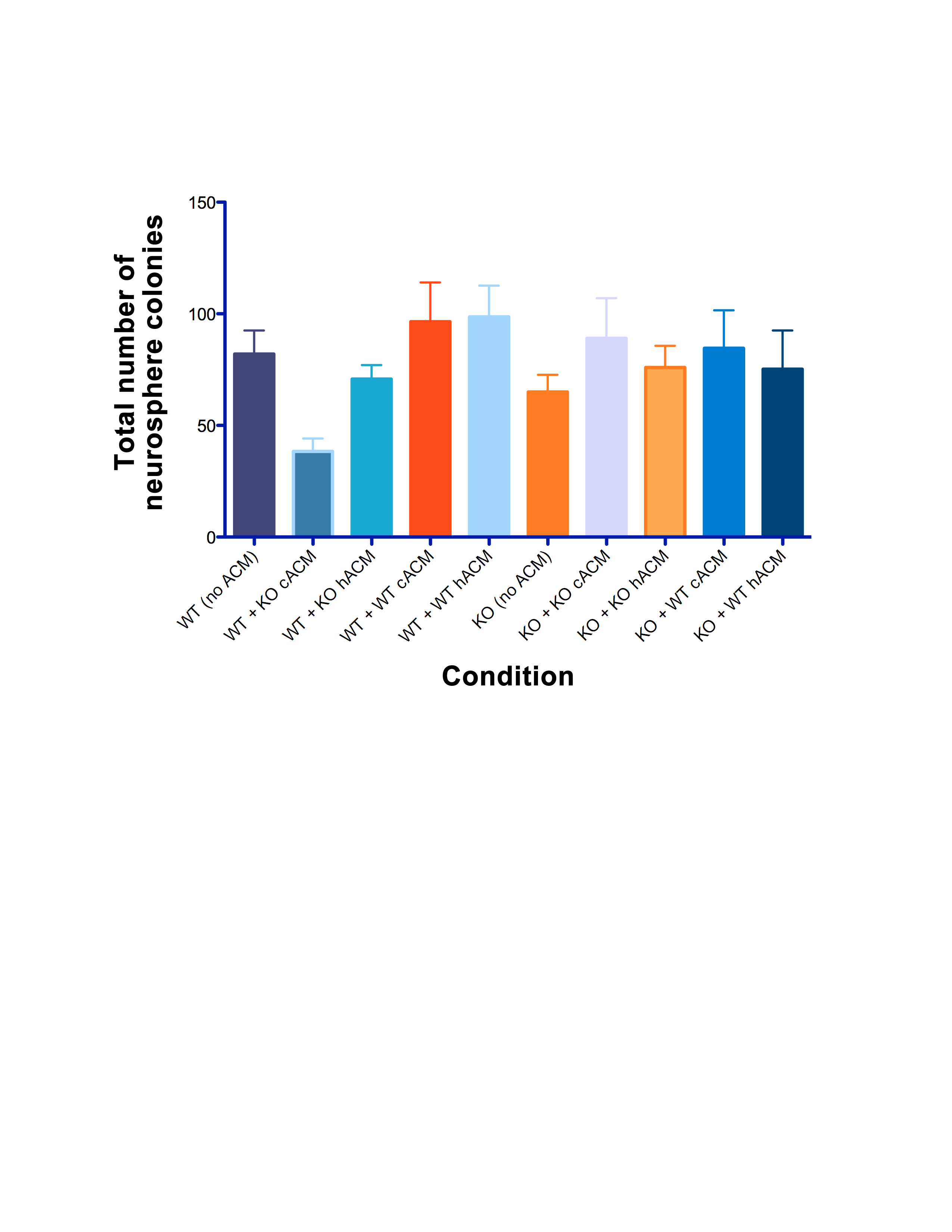

Supplement: Supplementary file 1 [file Image_1.TIF]

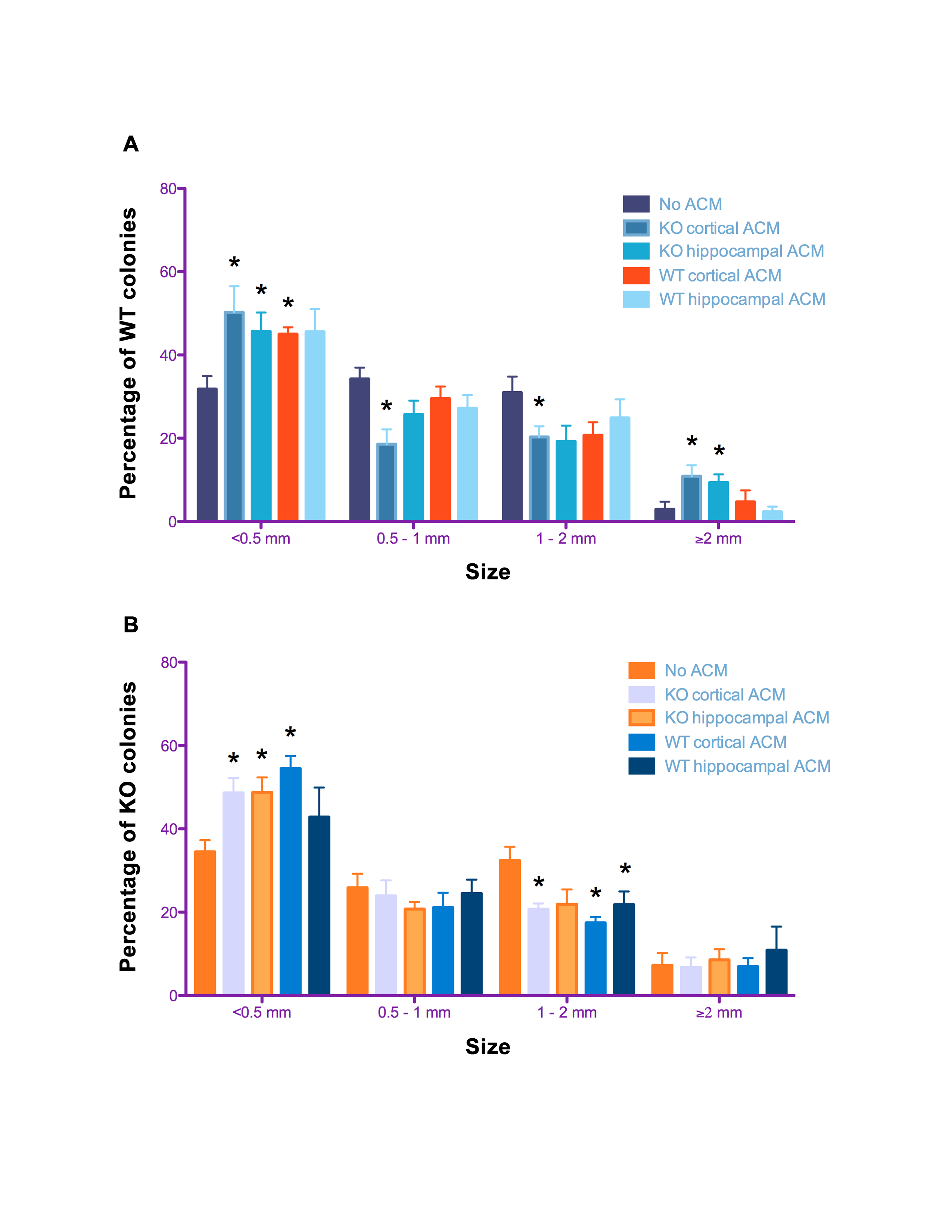

Supplement: Supplementary file 2 [file Image_2.TIF]
