# Supplementary material for: Astrocyte-Secreted Factors Selectively Alter Neural Stem and Progenitor Cell Proliferation in the Fragile X Mouse
Source: Front Cell Neurosci. 2016 May 18;10:126. doi: 10.3389/fncel.2016.00126 (PMC4870401; doi:10.3389/fncel.2016.00126)
Supplement: Supplementary file 3 [file Table_1.DOCX]

**Table S1.** Spots from cortical ACM with at least 1.5 fold difference relative to WT

| **Spot # on gel** | **Protein expression ratio relative to WT** |
| --- | --- |
| 1 | -1.6 |
| 2 | -4.9 |
| 3 | -2.4 |
| 4 | -1.7 |
| 5 | -1.9 |
| 6 | -3.1 |
| 7 | -3.6 |
| 8 | -2.4 |
| 9 | -1.7 |
| 10 | -1.7 |
| 11 | -2.1 |
| 12 | -3.4 |
| 13 | -2.1 |
| 14 | -3.1 |
| 15 | -1.7 |
| 16 | -4.8 |
| 17 | -3.6 |
| 18 | -2.3 |
| 19 | -1.6 |
| 20 | -2.2 |
| 21 | -2.1 |
| 22 | -2.2 |
| 23 | -3.1 |
| 24 | -4.5 |
| 25 | -4.2 |
| 26 | -3.7 |
| 27 | -5.2 |
| 28 | -10.6 |
| 29 | -1.9 |
| 30 | -2.7 |
| 31 | -3.5 |
| 32 | -2.3 |
| 33 | -12.9 |
| 34 | -3.5 |
| 35 | 1.5 |
| 36 | 1.9 |
| 37 | 1.6 |
