# Supplementary material for: Astrocyte-Secreted Factors Selectively Alter Neural Stem and Progenitor Cell Proliferation in the Fragile X Mouse
Source: Front Cell Neurosci. 2016 May 18;10:126. doi: 10.3389/fncel.2016.00126 (PMC4870401; doi:10.3389/fncel.2016.00126)
Supplement: Supplementary file 4 [file Table_2.DOCX]

**Table S2.** Spots from hippocampal ACM with at least 1.5 fold difference relative to WT

| **Spot # on gel** | **Protein expression ratio relative to WT** |
| --- | --- |
| 1 | 1.6 |
| 2 | 1.5 |
| 3 | 1.6 |
| 4 | 1.7 |
| 5 | 1.7 |
| 6 | 2.0 |
| 7 | 1.8 |
| 8 | 2.4 |
| 9 | 1.8 |
| 10 | -1.7 |
| 11 | -1.7 |
| 12 | 1.6 |
| 13 | 1.6 |
| 14 | -1.8 |
| 15 | -1.7 |
| 16 | 1.8 |
| 17 | 1.8 |
| 18 | 1.6 |
| 19 | 2.1 |
| 20 | 1.7 |
| 21 | 1.8 |
| 22 | -2.1 |
| 23 | 1.8 |
| 24 | -4.9 |
| 25 | -7.0 |
| 26 | -8.5 |
| 27 | -25.7 |
| 28 | -45.3 |
| 29 | -2.0 |
